# Sequence and Timing of Intracranial Changes in Cytomegalovirus in Pregnancy: A Case Report and Literature Review

**DOI:** 10.1155/2017/5928398

**Published:** 2017-04-10

**Authors:** Cynthia O'Sullivan, Shankari Arulkumaran, Lorin Lakasing, Eric Jauniaux, Karl Murphy

**Affiliations:** ^1^Department of Urology, Wellington Hospital, Wellington, New Zealand; ^2^Department of Obstetrics and Gynaecology, St. Mary's Hospital, Imperial College London NHS Trust, Praed Street, London W2 1NY, UK; ^3^Academic Department of Obstetrics and Gynaecology, Institute for Women's Health, University College London, 86-96 Chenies Mews, London WC1E 6HX, UK

## Abstract

Cytomegalovirus (CMV) is the most common cause of intrauterine infection, occurring in up to 2% of all live births. Most women are asymptomatic or experience nonspecific symptoms, which can lead to long-term sequelae in newborns including neurological impairment, hearing loss, and mental retardation. A 41-year-old woman (G6 P2), with a medical history of epilepsy, presented for her routine anomaly scan at 20 + 4/40. A single finding of echogenic bowel was noted on ultrasound which prompted a full investigation. A repeat ultrasound only five days later demonstrated progressive changes, which included bilateral ventriculomegaly with oedema of the posterior ventricular wall, periventricular hyperechogenicity, and enlargement of the cisterna magna. CMV DNA was detected at amniocentesis. Ultrasound findings are not diagnostic for CMV with only 11–15% of at-risk fetuses being identified. Unfortunately, these findings may be the only indication of an abnormality. There is a well-documented lack of awareness surrounding CMV and screening is not routinely offered. Given the risk to the pregnancy of CMV and to subsequent pregnancies, simple education at the start of a pregnancy could significantly reduce the incidence of maternal CMV.

## 1. Introduction

CMV is the most common cause of intrauterine infection [1–3] with an incidence of 0.3–2% in all live born infants [1, 2, 4–6]. Infected women often present with nonspecific signs and symptoms, but the majority are asymptomatic [6]. Approximately 10% of newborns show symptoms at birth [1, 3–5] but this increases to 20–30% if their mothers were infected in the first trimester [7]. There is a 30% mortality in the affected infants and 90% will have long-term neurological impairment [4, 5]. Of the asymptomatic neonates, 10% will develop permanent sequelae, including hearing loss and mental retardation [1, 3–5].

CMV infection can be a result of a primary infection, a reinfection with a new strain, or a reactivation of the residing virus [1, 5, 7]. The rate of primary infection from mother to child is approximately 40% (range 24–75%), as opposed to 1–2.2% in the case of reinfection (secondary) with a new strain [6]. The impact of primary infection on the fetus however is more significant [1, 3, 4]. The latency between primary and secondary infection and the detection of these differences on ultrasound are still under debate [1]. It appears that the fetal infection is more common when maternal infection occurs later in pregnancy, but the severity of the infection is higher before 18 weeks of gestation [5]. Transmission of the virus to the fetus can, however, occur weeks after maternal primary or secondary infection [7].

The ultrasound findings in CMV are not diagnostic as many features are shared with other conditions [1]. In addition, some studies have demonstrated that these findings are only present in up to one-third of the cases [1]. Lazzarotto et al. [6], in fact, only found that ultrasound detected not more than 5% of the infected fetuses. Furthermore, new ultrasound features to help in the diagnosis of intrauterine CMV have not been identified yet [1]. As most countries do not offer universal CMV screening in pregnancy, ultrasound monitoring is important as it is currently the only way of monitoring and assessing the prognosis of a fetus infected by CMV [2–4, 6]. Around 50% of the infected fetuses after a primary infection will be affected and present with both extracerebral and cerebral features on ultrasound and up to one-third demonstrate cerebral features only [1].

Anti-CMV IgG avidity is currently the most reliable test to identify primary infection in a pregnant woman [6]. Low avidity indicates an acute or primary infection as opposed to high avidity which indicates no current or recent infection [6]. Anti-CMV IgG avidity performed before the 16–18th week of pregnancy will identify all women at risk to have an infected fetus with a reported 100% sensitivity. After 20 weeks of gestation, the sensitivity is drastically reduced [6]. Amniocentesis is recommended between 21 and 22 weeks of gestation; CMV is a slow replication virus and will take up to 6–9 weeks before it is excreted in the fetal urine, in amounts large enough to be detected in the amniotic fluid [1, 6]. Conducting an invasive procedure too early could in fact result in a false negative result [1].

Ultrasound is therefore a useful adjunct in predicting the likelihood of postnatal disease. It can also be used as a prognostic parameter as the positive predictive value of ultrasound increases 2-fold when results from invasive testing indicate fetal infection [1, 4]. We present an unusual case where ultrasound was essential to guide the diagnosis of congenital CMV.

## 2. Case Study

**Figure 1:**
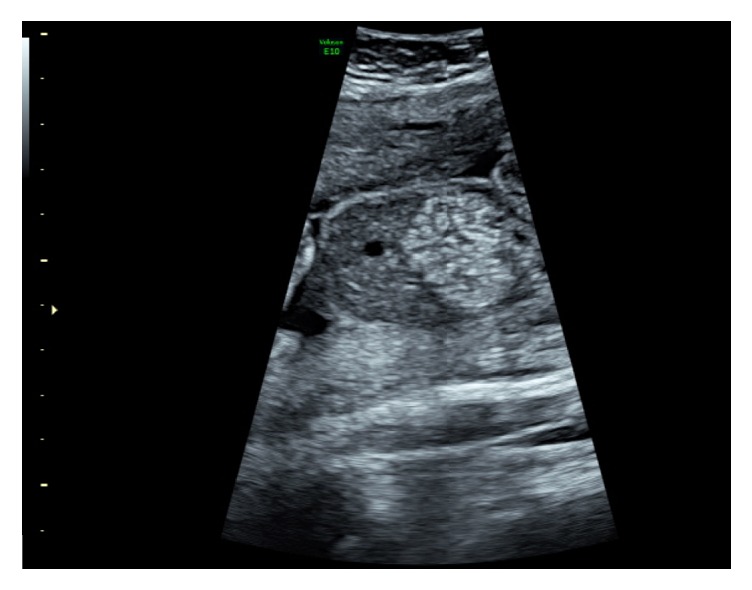
Echogenic bowel.

A 41-year-old Gravida 6, Para 2, presented in her first trimester with an unremarkable blood serology and a low combined screening risk for trisomy. Her obstetric history included 3 first-trimester miscarriages and a caesarean section and vacuum delivery, both at term. She was on medication for epilepsy but otherwise fit and well. Echogenic bowel was detected at her routine anomaly scan at 20 weeks and 4 days. She was referred to the Fetal Medicine Unit, where a repeat ultrasound, performed the following day, confirmed the findings of an isolated echogenic bowel with no other obvious structural anomalies (Figure 1). She was offered screening for a number of conditions known to be associated with echogenic bowel, including fetal aneuploidy, cytomegalovirus, toxoplasmosis, parvovirus, and cystic fibrosis. The couple declined an amniocentesis at this point.

The blood tests revealed that the couple were both carriers for the cystic fibrosis gene. In addition, the CMV IgG was positive, whilst the IgM was negative. This was checked retrospectively against her stored booking bloods, which revealed a positive IgG and IgM. The IgG antibody avidity was investigated and found to be low. Following these results, the couple proceeded with an amniocentesis at 22 weeks of gestation. The scan at this gestation still only revealed isolated echogenic bowel. The karyotype was normal but CMV DNA was detected in the amniotic fluid.

**Figure 2:**
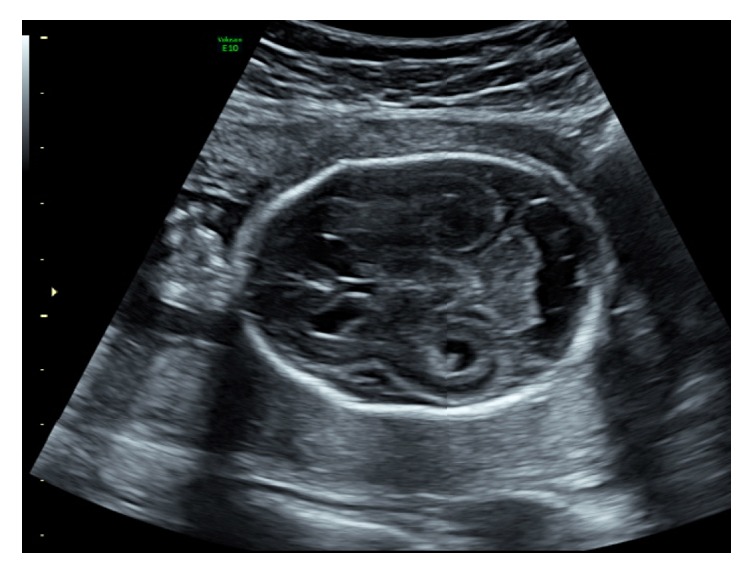
Bilateral ventriculomegaly with oedema of the posterior ventricular wall and periventricular hyperechogenicity.

**Figure 3:**
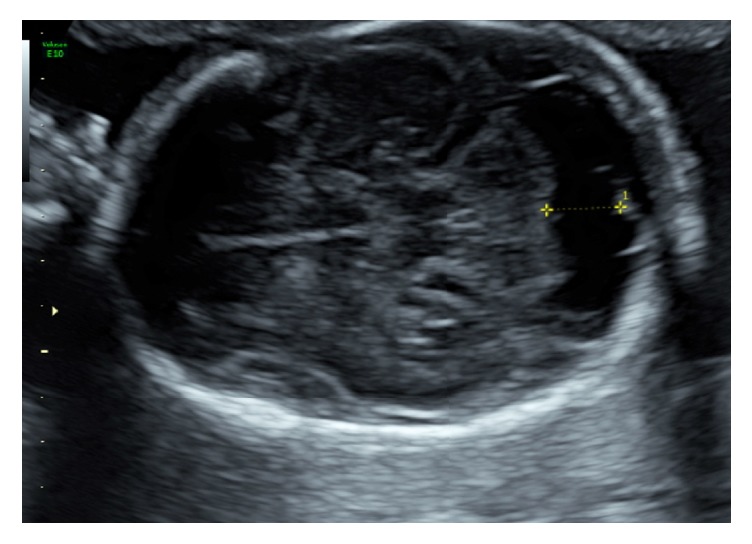
Enlargement of the cisterna magna.

However, only 5 days later, a further scan revealed new findings which included bilateral ventriculomegaly with oedema of the posterior ventricular wall, periventricular hyperechogenicity (Figure 2), and enlargement of the cisterna magna (Figure 3). The progressive changes on ultrasound indicated a poor prognosis for the fetus, and the couple terminated the pregnancy at 24 weeks of gestation. They declined a postmortem, but histopathology of the placenta demonstrated extensive lymphocyte infiltration with occasional CMV inclusions suggestive of chronic villitis due to the virus.

## 3. Discussion

### 3.1. Effect of Gestational Age

Information regarding the effect of gestational age on the outcome of the congenital infection is important as it is helpful in determining strategies for prevention, diagnosis, and treatment of infection in pregnancy [7]. As in our case, echogenic bowel and ventriculomegaly are the most common findings, but, despite this, they may only be identified in 11.8–15% of fetuses at risk [7]. Borderline ventriculomegaly is most common, but severe cases have been reported [4]. Lissencephaly is often described from 24 weeks of gestation and mega cisterna magna from 26 weeks of gestation [2, 4]. Other common features include periventricular hyperechogenicity, also identified in this case [2, 4]. Intraventricular synechia are seen in 50% of the cases, as well as intercranial calcifications in the periventricular area, brain parenchyma, cerebellum, and the corpus callosum [7]. Most report punctiform calcifications in the cerebellum, but linear calcifications have also been described [4]. In the transverse cerebellar plane, vermian defects can be identified [4]. Another classic feature of CMV in pregnancy is thalamic hyperechogenicity secondary to vasculitis, which is commonly referred to as the “candlestick sign” [2, 4]. Picone et al. [3] describe an occipital horn cyst, which has not been demonstrated in any other infective process, and they speculate that this may be unique to CMV infection in utero.

### 3.2. Pathogenesis

Understanding the development of the brain in utero is crucial to correlating findings seen on ultrasound scan with the exact time that maternal infection has occurred. The CMV virus, which is a double-stranded DNA virus, has a predisposition for the neuroblasts of the germinal matrix. This forms in the seventh week of gestation [8–10]. Infection before the eighth week of gestation leads to lissencephaly, when CMV interferes with neuronal migration [4], whereas infection between 18 and 24 weeks of gestation results in focal dysplastic cortices [2]. The cerebellum ends its formation by 18 weeks of gestation, and, therefore, the presence of cerebellar anomalies is suggestive of maternal infection prior to this [2]. Periventricular cysts and germinal matrix necrosis are seen in the second trimester, whereas fetuses with normal gyral patterns and periventricular echogenicities are probably injured in the third trimester [2]. Neuronal growth is complete by 26 weeks of gestation, so infections later in pregnancy have a little effect [10]. Similar to our case, Malinger et al. [2] described one case of rapid changes occurring within a week, from solely intraventricular adhesions to periventricular irregular patterns in the germinal matrix adjacent to the occipital horns.

Subependymal cystic lesions or calcifications, which develop during the second trimester, are thought to be the result of the necrotizing inflammatory effect of CMV on the subependymal germinal matrix of the lateral ventricles [2, 8]. Furthermore, it is thought that the scattered cerebral calcifications seen in the basal ganglia and thalami may correlate to the severity of the disease [8]. The encephaloclastic effect of the virus disturbs the cell proliferation in the developing brain, causing brain atrophy, and dilated ventricles may be seen as a result. Ventriculomegaly may also be a result of vasculitis or inflammatory exudates obstructing the flow of cerebrospinal fluid [8, 9].

### 3.3. Prognosis

It appears that children with congenital CMV infection were more likely to have serious sequelae if their mothers were infected earlier in the pregnancy. 23% of the infants were symptomatic when CMV infection occurred in the first trimester, compared to 11.4% in mothers infected after the first trimester [7]. There was also a trend towards greater abnormal neurological ultrasound findings being identified in those mothers infected in the first trimester. 26% of the cases of CMV diagnosed in pregnancy showed abnormal findings if the mothers were infected before 20 weeks of gestation, as opposed to 6.2% after 20 weeks of gestation [11]. Romanelli et al. [5] did not find any correlation between ultrasound findings and fetal infection, and Guerra et al. [1] stated that only fetuses with a severe disease will demonstrate obvious ultrasound abnormalities. The authors, however, do agree with Malinger et al. [2] that any combination of features on ultrasound do indicate a poorer prognosis. Unfortunately, because of the pathophysiology of CMV, several weeks can elapse before ultrasound features become obvious, and sometimes this may be the case until the third trimester [1]. It is therefore recognised that fetal cerebral features on ultrasound may not appear until significantly after maternal infection has occurred [4].

The findings on fetal MRI are fairly well correlated with ultrasound features [5], but the MRI is considered better in detecting abnormal gyration [1]. It may be useful if ultrasound imaging is inconclusive or difficult secondary to an unfavourable fetal lie or maternal habitus [12]. When there are no cerebral findings, MRI is not indicated [3], and, in our case, the findings were so severe, and MRI would not have changed management.

### 3.4. Subsequent Pregnancies

When counseling the parents following a primary CMV infection, it is important to provide information regarding the risks of CMV in subsequent pregnancies; the transmission rate can vary from 0.2% to 7% [13–15]. Of those infected, up to 8% of the infants may have neurological sequelae [15]. Reinfection with a different strain of CMV can occur in up to 62% of the seropositive mothers [16]. This is complicated by the fact that infected infants born to mothers with recurrent CMV infection are rarely symptomatic at birth making it more difficult to diagnose clinically [14, 15]. Transmission of CMV often occurs through saliva and the urine of infected children; therefore, although the risk cannot be eliminated, education on hygiene and behavioural measures should be provided to all women regardless of the serological status [6].

### 3.5. Prevention and Treatment

It is important to prevent CMV to reduce the burden of morbidity associated with congenital infection [12]. However, neither the Royal College of Obstetricians and Gynaecologists nor the National Collaborating Centre for Women's and Children's Health recommends routine CMV screening of all pregnant women. At present, treatment has been focused on reducing the adverse outcomes in the infected children after birth [12]. Studies using Ganciclovir following birth to prevent late-onset hearing loss have been controversial, as there have been high losses to follow-up and serious side effects, including haematological toxicity, have been demonstrated [12]. Oral Valaciclovir has been used to try and treat symptomatic CMV in utero, but no significant benefits with treatment have been demonstrated [12]. Similarly, research has been carried out into the antenatal administration of hyperimmune globulin therapy to try and reduce either the rate of transmission or the severity of the disease in an infected fetus [5]. This preparation uses pooled human plasma from screened donors to provide a therapeutic alternative for congenital CMV to termination of pregnancy or conservative management, whilst avoiding the potential fetal toxicity of antivirals such as Ganciclovir [12]. However, its efficacy has not been proven in any randomised controlled trials, and there have not been any long-term follow-up studies to look at adverse sequelae to the neonates in mothers who have been treated with the immunoglobulin [1, 12].

### 3.6. Awareness Campaigns

There is a well-documented lack of awareness surrounding CMV; Cannon et al. [17] found that only 7% of men and 13% of women had heard of CMV infection and mean to prevent the spread of the disease [18, 19]. Due to the asymptomatic nature of CMV infection and lack of public awareness, the mechanisms of prevention are currently centred around education and hygiene. Studies have shown that simple education at the start of a pregnancy could significantly reduce the incidence of maternal CMV [18, 20, 21]. CMV Action [22] is one of the nonprofit organisations based in the United Kingdom to raise the public's awareness of congenital CMV and publicise any new CMV research findings through their website and social media. They offer support to families that may have been affected by the condition and are working with medical professionals with an interest in the field.
